# Quantitative Gait and Balance Outcomes for Ataxia Trials: Consensus Recommendations by the Ataxia Global Initiative Working Group on Digital-Motor Biomarkers

**DOI:** 10.1007/s12311-023-01625-2

**Published:** 2023-11-13

**Authors:** Winfried Ilg, Sarah Milne, Tanja Schmitz-Hübsch, Lisa Alcock, Lukas Beichert, Enrico Bertini, Norlinah Mohamed Ibrahim, Helen Dawes, Christopher M. Gomez, Hasmet Hanagasi, Kirsi M. Kinnunen, Martina Minnerop, Andrea H. Németh, Jane Newman, Yi Shiau Ng, Clara Rentz, Bedia Samanci, Vrutangkumar V. Shah, Susanna Summa, Gessica Vasco, James McNames, Fay B. Horak, on behalf of the Ataxia Global Initiative (AGI) working group Digital Motor Biomarkers

**Affiliations:** 1 Section Computational Sensomotorics, Hertie Institute for Clinical Brain Research, Otfried-Müller-Straße 25, 72076 Tübingen, Germany; 2 Centre for Integrative Neuroscience (CIN), Tübingen, Germany; 3 Bruce Lefroy Centre for Genetic Health Research, Murdoch Children’s Research Institute, Parkville, VIC Australia; 4 Department of Paediatrics, Melbourne University, Melbourne, VIC Australia; 5 Physiotherapy Department, Monash Health, Clayton, VIC Australia; 6 School of Primary and Allied Health Care, Monash University, Frankston, VIC Australia; 7 Experimental and Clinical Research Center, a cooperation of Max-Delbrueck Center for Molecular Medicine and Charité, Universitätsmedizin Berlin, Berlin, Germany; 8 Neuroscience Clinical Research Center, Charité - Universitätsmedizin Berlin, Berlin, Germany; 9 Translational and Clinical Research Institute, Faculty of Medical Sciences, Newcastle University, Newcastle upon Tyne, UK; 10 NIHR Newcastle Biomedical Research Centre, Newcastle University, Newcastle upon Tyne, UK; 11 Department of Neurodegenerative Diseases and Hertie-Institute for Clinical Brain Research, University of Tübingen, Tübingen, Germany; 12 Research Unit of Neuromuscular and Neurodegenerative Disorders, Bambino Gesu’ Children’s Research Hospital, IRCCS, Rome, Italy; 13 Faculty of Medicine, Universiti Kebangsaan Malaysia, Kuala Lumpur, Malaysia; 14 NIHR Exeter BRC, College of Medicine and Health, University of Exeter, Exeter, UK; 15 Department of Neurology, The University of Chicago, Chicago, USA; 16 Behavioral Neurology and Movement Disorders Unit, Department of Neurology, Istanbul Faculty of Medicine, Istanbul University, Istanbul, Turkey; 17 IXICO, London, UK; 18 Institute of Neuroscience and Medicine (INM-1)), Research Centre Juelich, Juelich, Germany; 19 Institute of Clinical Neuroscience and Medical Psychology, Medical Faculty & University Hospital Düsseldorf, Heinrich Heine University Düsseldorf, Düsseldorf, Germany; 20 Department of Neurology, Center for Movement Disorders and Neuromodulation, Medical Faculty & University Hospital Düsseldorf, Heinrich Heine University Düsseldorf, Düsseldorf, Germany; 21 Nuffield Department of Clinical Neurosciences, University of Oxford, Oxford, UK; 22 Wellcome Centre for Mitochondrial Research, Newcastle University, Newcastle upon Tyne, UK; 23 Department of Neurology, Oregon Health & Science University, Portland, OR USA; 24 APDM Precision Motion, Clario, Portland, OR USA; 25 Movement Analysis and Robotics Laboratory (MARLab), Neurorehabilitation Unit, Neurological Science and Neurorehabilitation Area, Bambino Gesù Children’s Hospital, IRCCS, Rome, Italy; 26 Department of Electrical and Computer Engineering, Portland State University, Portland, OR USA

**Keywords:** Cerebellar ataxia, Gait and posture, Digital motor performance marker

## Abstract

With disease-modifying drugs on the horizon for degenerative ataxias, ecologically valid, finely granulated, digital health measures are highly warranted to augment clinical and patient-reported outcome measures. Gait and balance disturbances most often present as the first signs of degenerative cerebellar ataxia and are the most reported disabling features in disease progression. Thus, digital gait and balance measures constitute promising and relevant performance outcomes for clinical trials.

This narrative review with embedded consensus will describe evidence for the sensitivity of digital gait and balance measures for evaluating ataxia severity and progression, propose a consensus protocol for establishing gait and balance metrics in natural history studies and clinical trials, and discuss relevant issues for their use as performance outcomes.

**Supplementary Information:**

The online version contains supplementary material available at 10.1007/s12311-023-01625-2.

## Introduction

Based on the recent success of preclinical studies in genetic ataxias, and with several clinical trials currently active, targeted, disease-modifying therapies are on the horizon for spinocerebellar ataxias (SCAs) such as SCA1, 2, 3, and 6 and Friedreich’s ataxia [1–4]. To perform such clinical trials, there is a critical need for markers evaluating therapeutic outcomes [1, 5, 6]. As the genetic ataxias are rare disorders and current clinical and patient-reported measures demonstrate limited responsiveness, it is crucial to identify more sensitive markers of early disease and individual disease progression to enable trials with smaller sample sizes [5–8].

Gait and balance disturbances often represent the earliest signs of degenerative ataxia [9–11] and are reported by people with ataxia as one of the most disabling features affecting functional mobility as the disease progresses [12–15]. Thus, measures of gait and balance impairments qualify as both ecologically valid markers of progression and treatment response markers in future clinical trials.

Variability measures of ataxic gait and postural sway in stance have been shown to be strongly related to ataxia severity in multiple cross-sectional studies (reviews in [16–19]), including sensitivity in pre-ataxic disease stages [20–23]. The pre-ataxic stage includes carriers of SCA mutations before the manifestation of clinical ataxia symptoms defined by a SARA score below the threshold of 3 points [8, 24]. Digital gait and balance measures are now considered promising candidate outcomes for clinical trials and have been integrated into observational trials to yield further evidence [5, 25, 26].

In addition to cross-sectional sensitivity to early ataxia, clinical trials need objective measures that are capable of reflecting the slowing of disease progression within a reasonable study period (e.g. within 1–2 years). Hence, longitudinal, rather than cross-sectional, studies of gait and balance are needed to determine trajectories of digital measures, alongside clinical measures (e.g. the SARA score) and underlying biomarkers of disease progression. Given the limited number of people with these rare diseases, pooling of patient populations in multicenter, natural history studies will be most effective. This calls for highly standardized procedures of assessment [10, 27].

Measures characterizing the temporal and spatial variability of gait patterns in ataxia have been examined using a wide range of recording technologies (see for reviews [16, 18, 28]), from marker-based capturing systems as gold standard [20, 29, 30], electronic gait mats [22, 31–33], camera-based systems [34, 35] and body-worn inertial measurement units (IMUs) [21, 23, 33, 36–38]. Variability of gait can be measured both in the clinic/laboratory via active monitoring of prescribed tasks and passive monitoring during daily life. We will review the advantages and challenges of different motion recording technologies. For suitability in multicenter clinical trials, it is important to consider aspects like cost, feasibility without a dedicated gait laboratory or specialist staff, time required to prepare for the measurements, need of expertise in data processing, limitations in the spatial measurement range as well as the potential to characterize gait in daily life.

Based on this assessment and our current knowledge on sensitive gait and balance measures in ataxia, we present an evidence-informed proposal for: (i) a common protocol of gait and balance tests for natural history studies, (ii) sensitive gait and balance measures to be calculated and (iii) recommended data acquisition technology. With this consensus proposal, we aim to stimulate further research within the ataxia community on digital gait and balance measures to meet the requirements of future clinical trials.

## Aims and Objectives

The aim of this manuscript is to:

1. *Summarize patient-reported mobility impairments as well as the associated decline in quality of life resulting from ataxia.*
2. *Review evidence for specific measures of walking function and standing balance for use in ataxias.* Digital outcome measures should fulfill the following clinimetrics:
  - Sensitivity/specificity to premanifest and mild-moderate ataxia;
  - Concurrent validity (e.g. significant correlations with clinical rating scales);
  - Sensitivity to change over time (longitudinal) and in response to therapy (interventions);
  - Test-retest reliability and minimal detectable change (MDC) necessary to detect subtle changes;
  - Meaningfulness to people with ataxia.
3. *Recommend a common protocol for multicenter, natural history studies to support the inclusion of digital gait and stance tasks as useful outcomes for clinical intervention trials on ataxia.* Standardizing the motor tasks and harmonizing the protocols, instructions, metrics, and technologies is important for pooling data on rare forms of ataxia for regulatory approval of digital outcomes for future clinical trials.
4. *Identify the remaining necessary steps to implement digital gait and balance outcome measures in the context of future intervention trials.*

Consensus has been reached in three rounds as follows: (1) Collecting relevant topics and requirements towards trial-readiness of digital motor performance measures; (2) Discussing content issues related to motor tests, measures and recording technologies; (3) Drafting and reviewing the manuscript.

## Patient-Reported Impairments in Mobility and Associated Decline of Quality of Life

There is ample evidence from patient-reported outcome measures [12–14, 39] and questionnaires on quality of life [40–44] that walking and balance are central factors of functional (im)mobility in the disease-related decline in quality of life of people with ataxia. To measure the impact of ataxia on quality of life, many studies use the EQ-5D [45], a generic, standardized measure of health-related quality of life, which assesses health status across five dimensions (mobility, self-care, usual activities, pain/discomfort and anxiety/depression). In several studies of SCAs [40–42], the most frequently patient-reported problems were with mobility (“I have moderate/severe problems walking”), even in mildly to moderately affected SCA participants [42]. In addition, the mobility dimension revealed the largest progression slopes in long-term evolution of ataxia [43].

Two FDA (Food and Drug Administration) “Voice of the Patient” meetings have confirmed that people with ataxia feel that walking difficulty is the biggest challenge in daily living [12, 13]. In addition, most of them identified “Lack of balance” as the ataxia symptom with the greatest impact on daily life [13]. “My balance and coordination are affected so I leave class early to avoid the crowded halls”, “SCA2 affects my balance and coordination making me look drunk while walking”, “I ended up losing my balance and falling” [13]. Similarly, balance problems were the most commonly reported problem affecting daily quality of life in Friedreich's ataxia [12, 14, 15].

The importance of gait and mobility limitations is also reflected in the recent development of the PROM-Ataxia scale, in which 147 people with ataxia described their disease symptoms and the associated limitations in daily life [39]. Impaired balance and gait were the most frequently mentioned symptoms in both the physical domain and the domain of daily activities [39].

## Digital Gait and Balance Measures Quantifying Ataxia

### Clinical Gait Assessment

Clinically, ataxic gait is typically characterized by unstable, stumbling walking, increased step width and high gait variability [46–49]. The characteristic high variability of walking patterns in people with ataxia are thought to result from the complex interaction between cerebellar-induced deficits in balance control and multi-joint coordination, the compensatory/safety strategies used, and inaccurate postural adjustments to apparent losses of balance [50].

Accordingly, there is broad consensus [10, 16–18, 21, 50, 51] that the most striking and distinctive features of ataxic gait are the high variabilities in spatial and temporal metrics (e.g. stride-to-stride variability in length, stride width, stride duration). Although people with ataxia may also show altered pace with slow gait velocity, small step length, long double-support phase and wide step width, these metrics are less specific and sensitive to ataxia and may reflect compensatory strategies and general slowing of gait, e.g. to avoid losing balance and falling, rather than primary cerebellar deficits in control of gait [18].

Gait variability measures have been shown to be sensitive and specific for ataxia, as well as significantly related to clinical ratings of ataxia severity, such as the ICARS [52], SARA [24], BARS [53] and FARS [54] in multiple cross-sectional studies [21–23, 29–31, 34, 36, 37, 50, 55–58] (see Table 1 for an overview).

**Table 1:** Overview of studies on quantifying on cerebellar ataxic gait. *SCA*, autosomal-dominant spinocerebellar ataxia of defined genetic type; *CA*, cerebellar ataxia; *DCA*, degenerative cerebellar ataxia; *FRDA*, Friedreich’s ataxia; *MSA-C*, multiple system atrophy type C; *HC*, healthy controls; *SARA*, scale for the assessment and rating of ataxia; *BARS*, Brief Ataxia Rating Scale

Study	Population	Aim	Protocol	Recording system	Primary gait measures
Palliyath 1998 [59]	10 ataxic participants with cerebellar damage	Describe the gait pattern in ataxia	6–10 trials * 2.5 m capture volume	Infrared-cameras, marker-based	Variability in knee and ankle joint angles, step length,
Ilg 2007 [55]	13 participants CA	Identify parameters related to balance vs interlimb coordination	8 m walkway	Infrared-cameras, marker-based	Variability in intra-limb temporal coordination
Morton 2003 [60]	20 participants CA	Determine relationship of balance and gait impairments	8 m walkway	Infrared-cameras, marker-based	Variability of stride length, stride width
Serrao 2012 [50]	16 participants with SCA1, SCA2, FRDA	Characterize ataxic gait	10 m walkway with 4m recording path	Infrared-cameras, marker-based	Variability in step parameters and joint angle trajectories
Serrao 2017 [61]	12 participants with deg. cerebellar ataxia	Identify longitudinal change over 2 and 4 years	6 * 10 m walkway	Infrared-cameras, marker-based	Step length, step variability
Schniepp 2012 [32]	40 participants with CA	Identify influence of gait speed on gait variability	6.7-m-long instrumented mat	*GAITR*ite	Variability of step time
Rochester 2014 [22]	24 participants with SCA6 (6 pre-ataxic; 18 ataxic)	Identify pre-ataxic gait impairments	7-m-long instrumented mat	*GAITR*ite	Variability of step time and step length
Schmitz-Hübsch 2016 [33]	12 participants with SCA14 and 9 HC	Comparing accuracy and repeatability of two methods of gait analysis	5.1-m-long instrumented mat	*GAITR*ite, Inertial sensors (#3)	Spatial gait measures in average and variability
Milne 2021 [62]	61 participants with FRDA	Longitudinal change in FRDA	7-m-long instrumented mat	*GAITR*ite	Variability of step time, speed
Summa 2020 [34]	31 young participants with CA	Validation of low-cost system for gait assessment in children	10 *3 m pathway	Kinect	Speed, stride length
Ilg 2022 [25]	28 participants with SCA3	Longitudinal change in SCA3	5 * 8 m walkway	Multi-Kinect system	Variability of step length, trunk sway lateral amplitude
Hickey 2006 [57]	22 participants with SCA6	Validity of accelerometer to quantify ataxic gait	7 m walkway	Inertial sensors (#1)	Speed, step length
Terayama 2018 [63]	14 participants with SCA	Correlation with SARA gait item	5 m walkway	Accelerometer (#1)	Step length, step-width variation
Phan 2019 [64]	29 participants with CA	Quantify ataxia-related changes in slow, preferred, fast speed	Slow, straight walking	Inertial sensors (#3)	Lateral amplitude of trunk sway
Shirai 2019 [65]	25 participants with SCA	Sensitivity to longitudinal change	6 min walking on a 30 m walkway	Accelerometers (#2)	Lateral amplitude of trunk sway
Ilg 2020 [36]	43 participants with DCA	Cross-sectional sensitivity to ataxia severity	2 * 25 m, real life movement capture	Inertial sensors (#3)	Lateral step deviation, compound measure of spatial step variability
Shah 2021 [21]	163 participants with SCA types 1, 2, 3, 6 incl. 42 pre-ataxic	Cross-sectional sensitivity to ataxia severity	2 min on a 10 m parkour with 180° turns	Inertial sensors (#6)	Toe-out angle variability, double-support time variability
Velazquez-Perez 2021 [23]	30 participants with pre-ataxic SCA2	Identification of pre-ataxic gait changes	2 * 10 m	Inertial sensors (#6)	Variability in toe-off angle and toe-out angle
Castiglia 2022 [66]	24 participants with DCA	Quantify dynamic unbalance in cerebellar ataxia	30 m	Inertial sensor (#1)	Harmonic ratios
Zhou2022 [37]	14 participants with SCA types 1,2,3,6	Estimate the level of motor impairment in SCA	SARA gait item (10 m), BARS2 gait item (10 m)	Inertial sensors (#7)	Linear regression models using stride length variability, stride duration, cadence, etc.
Lee 2022 [67]	37 participants with ataxia (15 SCA, 4 MSA-C)	Analyse of gait sub-movements	5 m walkway	Inertial sensors (#2)	Regression models trained on features of movement elements estimated the BARS
Gouelle 2021 [68]	19 participants with FRDA	Development of compound scores	6-m-long instrumented mat	Zeno, ProtoKinetics	Variability score, clobal ambulation score
Kadirvelu 2023 [69]	9 participants with FRDA	Prediction of longitudinal change	8 m walkway	Inertial sensors (#17)	Correlation to longitudinal SARA change
Vasco 2016 [70]	11 participants with FRDA	Analysis of longitudinal change	10 m walkway	Infrared-cameras, marker-based; force plate	Knee peak angle as sensitive measures for longitudinal change
Stephenson2015 [71]	8 participants with FRDA	Relationship between gait/balance measures and clinical scales	7-m-long instrumented mat	*GAITR*ite	Stride length variability increased for comfortable and fast walking
Zesiewicz 2017 [72]	8 participants with FRDA	Analysis of longitudinal change	7-m-long instrumented mat	*GAITR*ite	Gait speed showed changes after 12 and 24 months for comfortable and fast conditions
Eklund 2023 [73]	43 participants with ataxia (29 SCA, 6 MSA-C)	Relationship between ankle submovements and patient-reported gait impairment	real life movement capture	Inertial sensor (#1)	Smaller, slower and less powerful ankle submovements in people with ataxia.

**Fig. 1:**
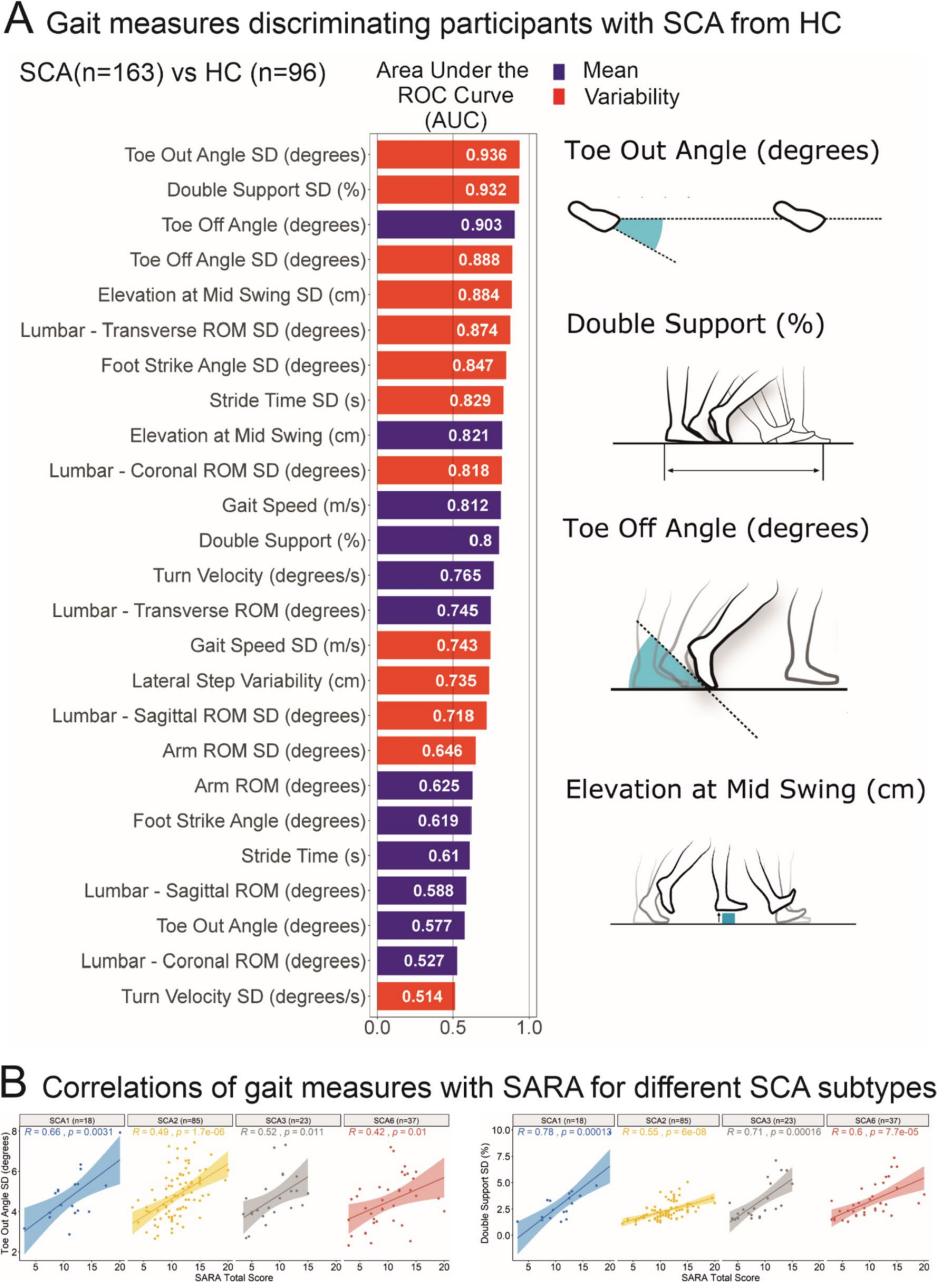
(A) Area under the ROC (receiver operating characteristic) Curve (AUC) in descending order for each gait measure discriminating people with spinocerebellar ataxia (SCA) from healthy controls (HC). (B) Pearson correlation of the four most discriminative gait measures with clinical SARA scores related to the ataxia severity of each subtype of SCA 1,2,3 and 6 (adapted from [21])

Recently, in one of the largest studies (N=301) using a comprehensive set of gait measures from body-worn, inertial sensors, Shah et al. showed that variability measures were the most discriminative gait characteristics for mild-to-moderate SCA as well as for pre-ataxic SCA compared to healthy controls [21]. This study included measures of gait that depend on measuring foot orientation in space (such as toe-out angle, toe-off angle, the elevation of the feet at mid-swing), as well as trunk measures. The most sensitive and specific measures of gait variability, based on the Receiver-Operating Characteristic (ROC) area under the curve (AUC) to discriminate SCA from control performance, are summarized in Fig. 1A. Figure 1B shows significant correlations between these most sensitive gait variability measures and the SARA ataxia score [21].

**Fig. 2:**
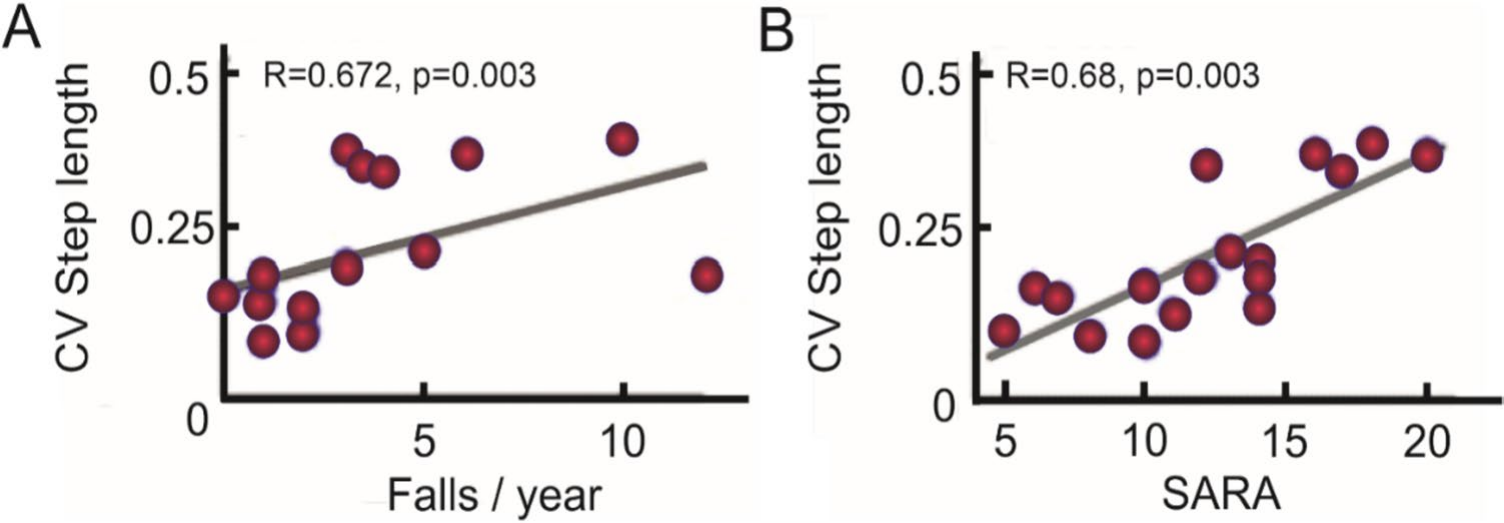
Correlations between the step length coefficient of variation (CV) and the falls/year (A) and SARA scores (B) in 17 ataxic participants. Pearson’s *R* coefficient (*R*) and significance (*p*) are reported (adapted from [74])

Besides the relationship with clinical ataxia severity, gait variability is also associated with patient-reported balance impairments. For example, gait variability (step length) is related to the number of reported falls [31, 66, 74] (see Fig. 2) and predicts future falls in cerebellar gait disorders [75]. In addition, gait variability (e.g. lateral step deviation) is correlated with the subjective confidence in daily life activities of balance, measured by the ABC-score (Activities-specific Balance Confidence) [36].

Unlike cerebellar ataxia in SCA, Friedreich's ataxia (FRDA) mainly affects afferent connections to the cerebellum, resulting in a combined cerebellar-sensory ataxia. Therefore, the most sensitive/specific gait characteristics may be different in this population. Serrao et al. [50] have conducted the only study comparing the gait characteristics of individuals with FRDA and SCA (SCA1 and SCA2) and found that gait pattern impairments were relatively consistent between groups with the exception of a shorter step length in FRDA. In cross-sectional studies in FRDA, stride length variability was correlated with balance outcomes [58, 71] during self-selected and fast walking speeds, whereas mean spatiotemporal parameters were correlated with falling frequency [76] and lower limb co-ordination [58]. In addition, double support time variability is sensitive to disease duration, balance decline, and ataxia (as measured by the FARS [58] and SARA [70]).

### Clinical Balance Assessment in Stance

Standing balance tasks allow the evaluation of ataxia-related, static balance impairments in a “purer” form, without the influence of locomotor dynamics or impairments in multi-joint coordination for goal-directed leg placement. Thus, measures of postural sway during quiet, unsupported stance (static posturography) provide a method to quantify the quality of postural (balance) control [77].

**Fig. 3:**
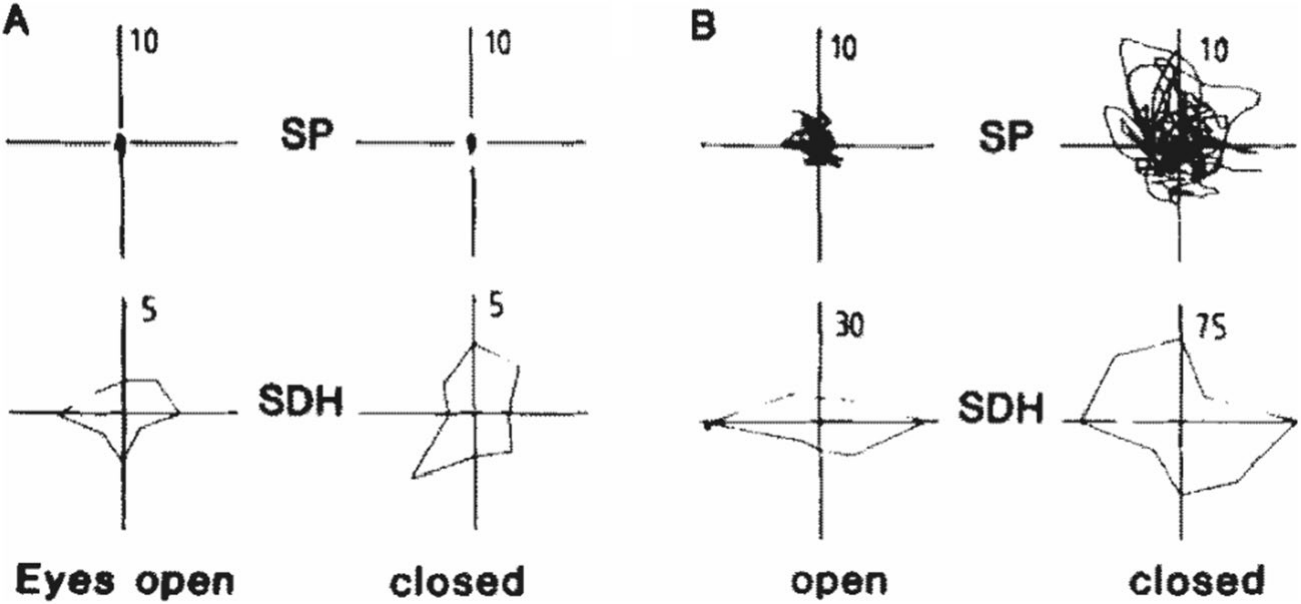
Recording of sway path (SP) in anteroposterior and lateral direction and the calculated sway direction histogram (SDH). (A) Normal subject. (B) Predominantly lateral sway and very large sway eyes closed, in a patient with Friedreich’s ataxia. Adapted from [80]

**Fig. 4:**
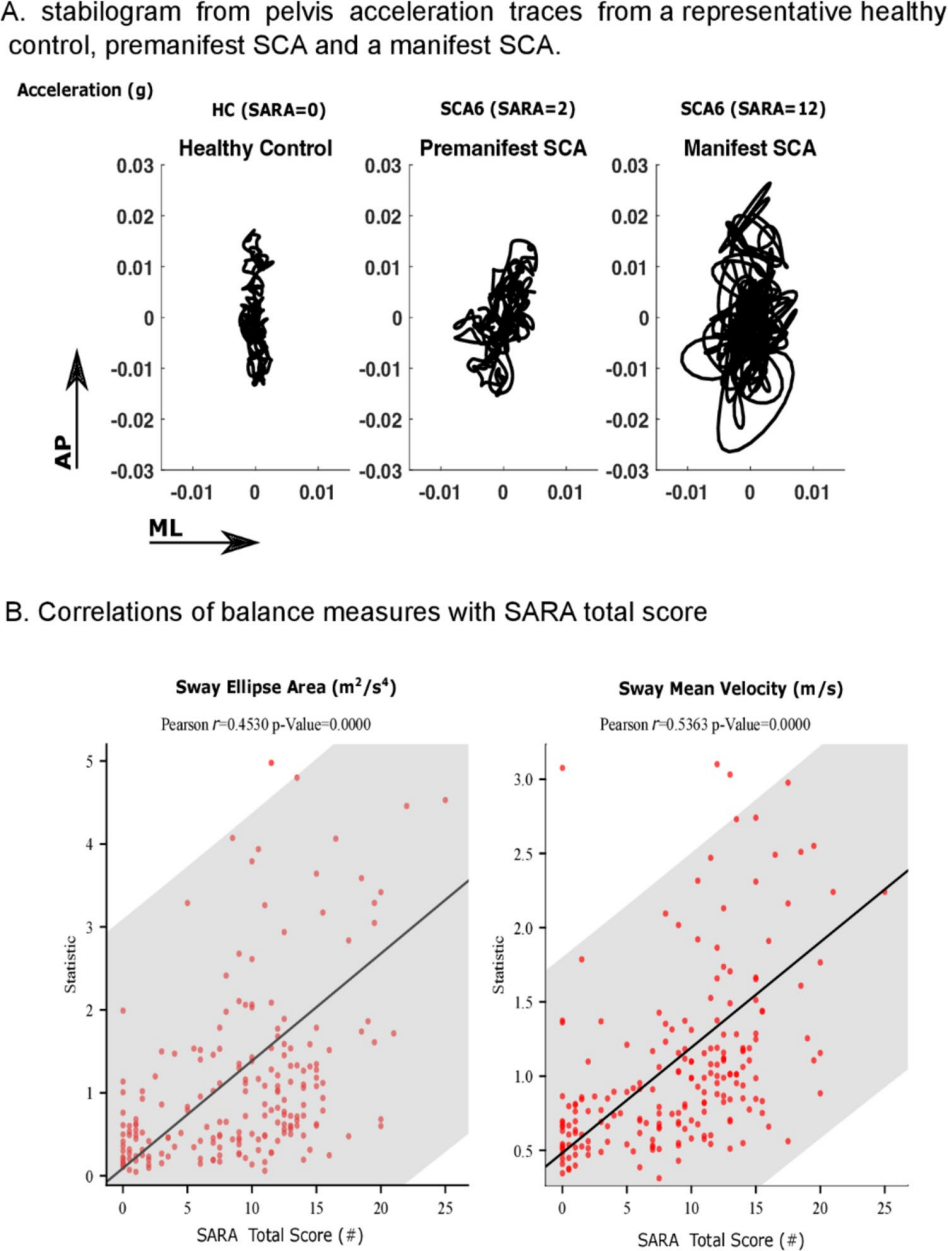
(A) Representative examples of representative statokinesiograms (postural sway path) during a 30-s, feet-together, eyes-open stance in a healthy control individual, an individual with pre-ataxic SCA6 and an individual with manifest SCA6. (B) Both sway ellipse area and sway mean velocity are correlated with severity of ataxia, as measured by the SARA in SCA 1,2,3 and 6 [85]

The cerebellum is responsible for integrating somatosensory, vestibular and visual inputs for control of balance and people with cerebellar ataxia become more dependent upon vision to control balance compared to controls [78–81]. Thus, postural balance tasks with eyes closed are particularly difficult for people with FRDA, likely associated with difficulty using proprioceptive feedback due to spinocerebellar degeneration [62, 82]. Postural stability with eyes closed is highly responsive to disease progression early in FRDA; however, 66% of independently ambulant individuals with FRDA cannot stand with eyes closed [62]. An early study by Diener et al. [83], compared postural sway eyes open (left) and eyes closed (right) for a healthy control and a person with FRDA (Fig. 3). Historically, studies have used a force plate to quantify postural sway as displacement of the body center of pressure, whereas more recent studies have used an inertial sensor placed near the body center of mass (Lumbar 2 level) to quantify anterior-posterior and mediolateral linear accelerations and/or angular velocities [84, 85] (Fig. 4). An overview of postural sway studies in people with ataxia is provided in Table 2.

**Table 2:** Overview of studies on quantifying posture control impairments in cerebellar ataxia. *EO*, eyes-open; *EC*, eyes-closed; *CA*, cerebellar ataxia; *SCA*, autosomal-dominant spinocerebellar ataxia of defined genetic type; *FRDA*, Friedreich’s ataxia; *MSA-C*, multiple system atrophy type C; *ADCA*, autosomal dominant ataxia of still undefined genetic cause; *OPCA*, olivopontocerebellar atrophy; *CCA*, cortical cerebellar atrophy; *HC*, healthy controls; *SARA*, scale for the assessment and rating of ataxia; *BARS*, Brief Ataxia Rating Scale

Study	Participants	Aim	Protocol/duration of recordings					Recording	Balance measures
			EO/ Feet Apart	EC/ Feet Apart	EO / Feet Closed	EC/ Feet Closed	EO/ Tandem		
Diener 1984 [81]	33 participants with CA 8 FRDA	Localize cerebellar lesions			60s	60s		Force plate	Path length, sway area
Asahina 1994 [86]	30 participants with CA	Usefulness of posturography	30s	30s				Force plate	Sway area, frequency
Gatev 1996 [87]	25 participants with CCA 9 OPCA	Sensory conditions	30s	30s				Force plate	Lateral and Anterior-posterior range of sway
Van de Warrenburg 2005 [88]	11 participants with CA, 11 HC	Usefulness of trunk gyro for ataxia	30s	30s	30s	30s		Gyroscope	Trunk angular velocity
Bunn 2013 [78]	17 participants with SCA 6	Stance stability and foot width	40s		40s			Force plate	Velocity
Matsushima 2015 [89]	Participants with SCA 13, 1, 2, 3, 6, ADCA, CCA, MSA-C	Usefulness of accel for standing	30s	30s	30s	30s		Accelerometers	RMS (root means square)
Ilg 2016 [20]	14 participants with pre-ataxic SCA 1,2,3,6, 9 SCA 1,2,3,6	Identify increased sway in pre-ataxic participants			30s	30s		3D Motion Capture	Path length
Nanetti 2017 [90]	9 participants with pre-ataxic SCA1	Longitudinal progression	30s	30s	30s	30s		Force Plate	Stability Index
Fleszar 2019 [91]	40 participants with CA	Audio- biofeedback			30s	30s		3D Motion Capture	Path length
Nguyen 2018 [92]	34 participants with CA 22 HC	Quantify postural stability			30s	30s		Accelerometers: Sternum	Area (RMS), Entropy
Liu 2020 [93]	62 participants with SCA3, 62 HC	Relation to clinical ataxia	30s	30s				Force plate	Range variability, Velocity
Galvao 2022 [94]	23 participants with SCA 3 102 HC	Ankle or Hip strategies	30s	30s				Force plate	Velocity CoP (center of pressure)
Velazquez-Perez 2021 [23]	30 participants with pre-ataxic SCA2, 30 HC	Identify pre-ataxic sway	30s		30s		30s	Lumbar,+ Sternum IMUs	Jerk, path length, velocity, area
Zhou 2022 [37]	14 participants with SCA,4 HC	Explore use in clinical test			10s			Lumbar IMU	Sway area and velocity
Shah 2022 [85]	101 participants with SCA1,2,3,6 40 pre-ataxic; 99 HC	Explore, which sway measures most sensitive	30s	30s	30s	30s		Lumbar + Sternum IMUs	Sway area and velocity
Ngo 2021 [95]	53 participants with CA	Digital and clinical assessments of Romberg's test.			30s	30s		Chest IMU	Recurrence rate, Multi-scale entropy, Harmonic ratios
Milne 2021 [62]	61 participants with FRDA	Change in postural stability over 12 months.	30s	30s				Force plate, Biodex Balance System	Limits of stability

### Gait and Stance Tasks with Increased Balance Challenge

#### Stance Tasks with Increased Balance Challenge

**Fig. 5:**
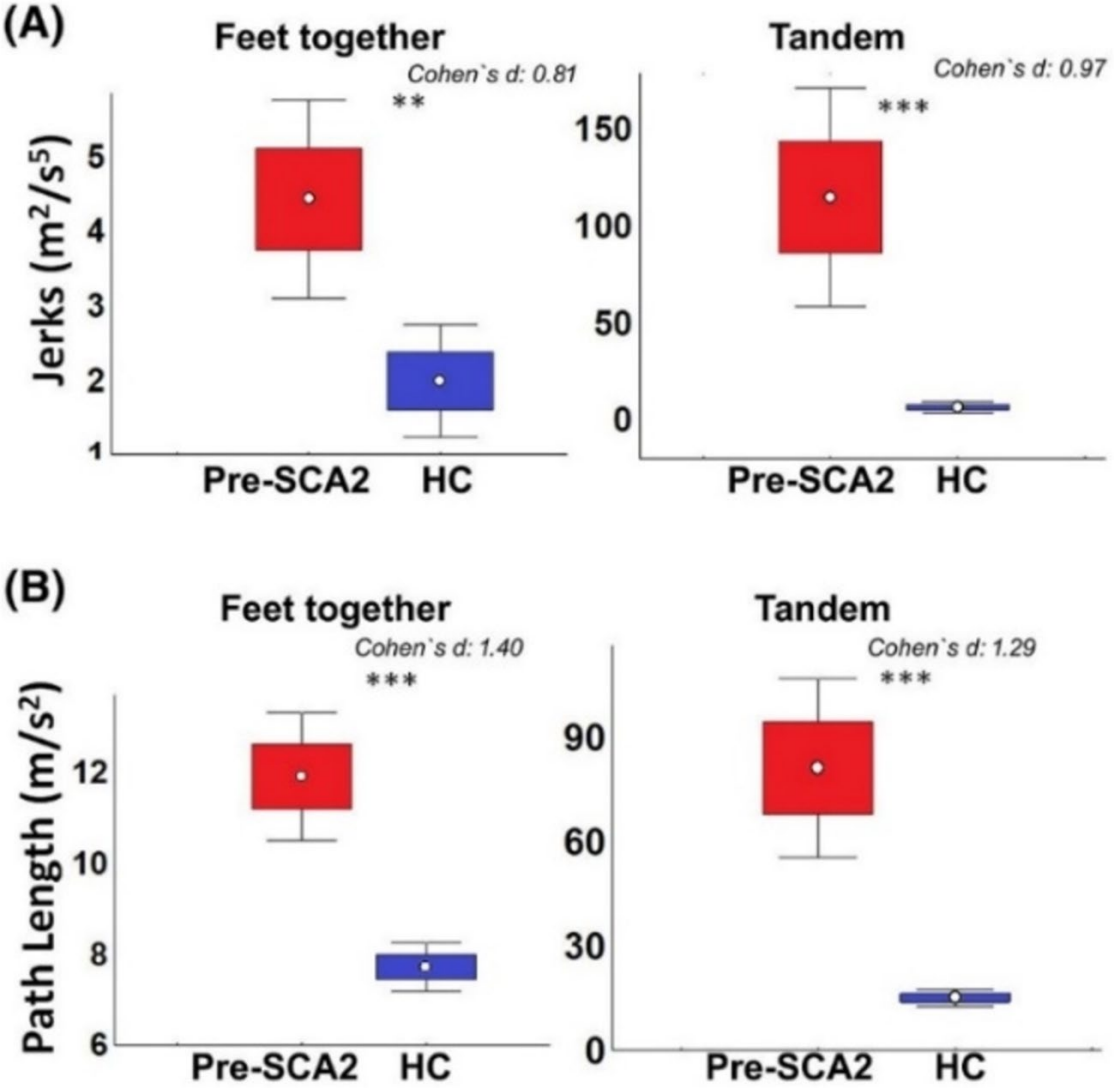
Postural sway abnormalities in pre-ataxic SCA2 participants (Pre-SCA2) in comparison to healthy participants (HC) for a stance task with feet together and for tandem stance. Shown are stance measures jerks (A) and Path Length (B). ns: *P* > 0.0013 (after Bonferroni correction); **, *P* < 0.005; ***, *P* < 0.0005; Adapted from [23]

**Fig. 6:**
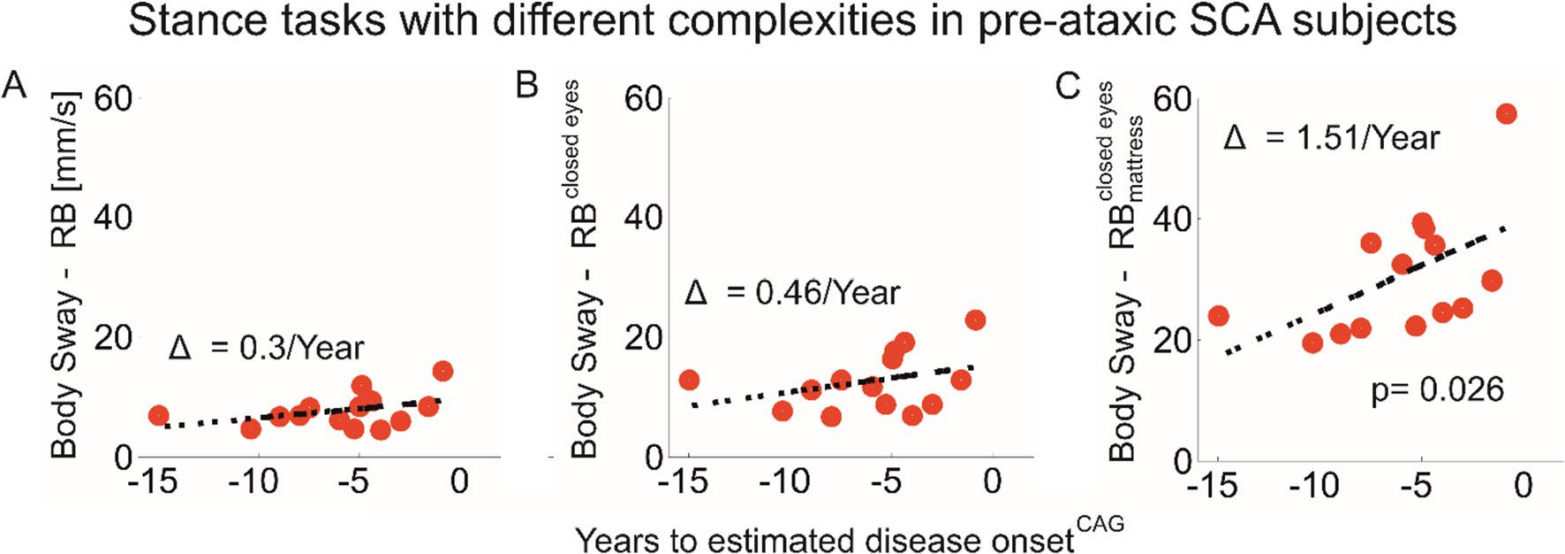
Relationship between body sway and estimated time to disease onset for pre-ataxic mutation carriers in different stance tasks. Shown are relationships for genetically-based estimates of onset according to [97]. Each circle represents one participant. Body sway (length of sway path) was determined in three different stance conditions: (A) feet closed (Romberg test, RB) and eyes open; (B) feet closed (Romberg) and eyes closed; (C) feet closed (Romberg test, RB) and eyes closed on a foam cushion (mattress). *P*-values indicate significant correlations between durations to estimated disease onset and body sway. Reprinted with permission from [20]

Postural sway characteristics may be more sensitive to detect early and pre-ataxic stages in more complex stance tasks, such as standing with feet together, on a foam surface or with feet in a tandem position or by eliminating visual feedback with eye closure [20, 23, 90, 96]. Pre-ataxic SCA2 participants showed a significantly larger postural sway and jerk both with feet together and in tandem positions compared to healthy controls (HCs) [23]. In fact, the more challenging the standing position, the stronger the relationship between postural sway and years to estimated disease onset in pre-ataxic SCA (types 1, 2, 3 and 6) [20]. Figure 5 shows the increased sensitivity of tandem stance compared to feet together stance in pre-ataxic participants compared to controls. Figure 6 shows the relationship between time to genetically-estimated disease onset [97] and postural sway [98] under various conditions: standing balance task with (A) eyes open, (B) eyes closed and (C) eyes closed on a foam cushion. Thus, studies on pre-ataxic SCA or people with ataxia close to disease onset should include stance tasks in more complex conditions. Adopting a wide stance on a firm surface with eyes open may not be challenging enough to identify impairments in standing balance in such populations.

#### Gait Tasks with Increased Balance Challenge

*Tandem gait* increases the demands on dynamic balance control and also on the accuracy of targeted foot movements and has therefore been shown to be very sensitive to detect mild cerebellar damage [51, 88, 99], including sub-clinical cerebellar deficits [29]. Recent studies revealed increases in body sway and stride time variability when walking in tandem in pre-ataxic SCA mutation carriers [20, 23] with correlations [20] to genetically determined estimations of disease onset [97]. The tandem gait is therefore a very sensitive test in the earliest stages of ataxia, but people with moderate to severe impairments are often unable to perform the test safely.

*Turning movements* represent a highly relevant component of everyday walking behaviour, since 35–45% of steps occur within turns [100]. Compared to straight walking, turning movements are more challenging in terms of dynamic balance [101–104], as they involve a stronger demand for anticipatory postural adjustments [105] and trunk-limb coordination strategies [106]. A recent study demonstrated that a measure — lateral velocity change (LVC) — which was used to quantify dynamic balance during turning, is sensitive to pre-ataxic stages and shows strong correlations to self-reported balance confidence in daily life as measured by the ABC-score (*r* > 0.65) [107]. Thus, dynamic balance measures while turning seem particularly sensitive for detecting subtle changes in ataxia and should be included in studies of pre-ataxic and early disease stages.

### Quantifying Walking Behaviour in Daily Life

There is legitimate concern that, despite the heightened potential for reproducibility, the assessment of gait in the clinic may not adequately reflect mobility function during daily life [108, 109]. Under a brief examination in the outpatient clinic, a person with ataxia may appear to walk and display balance better than caregivers report observing during their daily lives. Furthermore, a single, or sparsely spaced, measure of mobility cannot assess day-to-day or other clinically relevant windows of change, such as daily motor fluctuations or effects of fatigue.

Advances in wearable sensor technology enable not only standardized gait and stance assessments in clinical settings, but also allow recordings of gait behavior in everyday life.

Remote monitoring of mobility provides an extended period of observation in the more natural home setting, adding ecological validity to the observed measures.

**Fig. 7:**
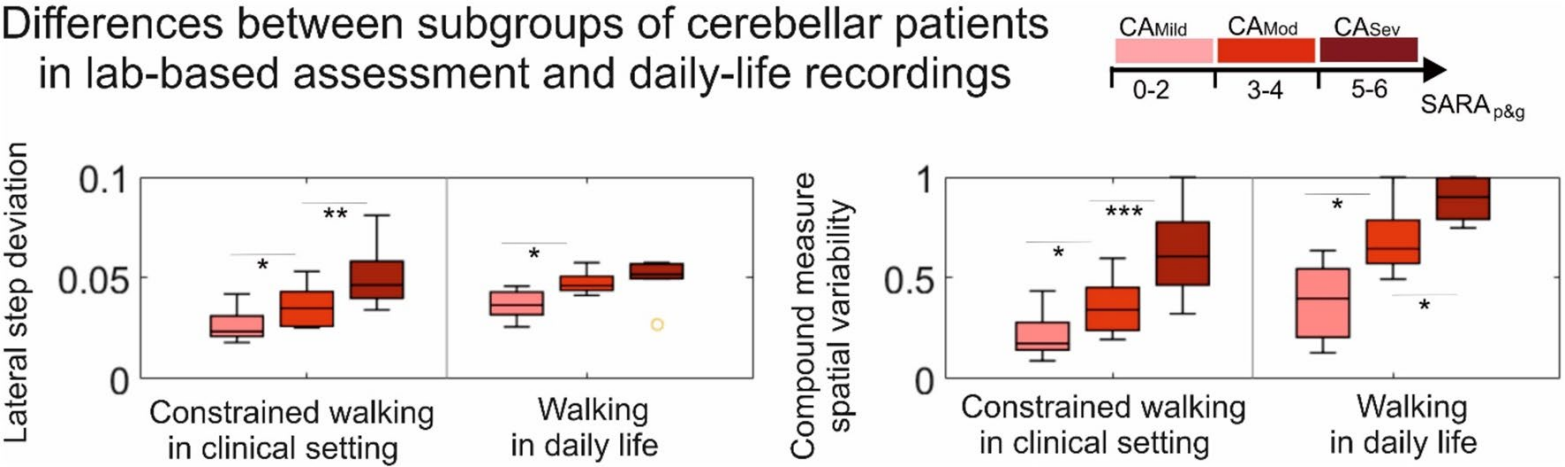
Differences between subgroups of participants with cerebellar ataxia (CA) stratified according to gait and posture ataxia severity as determined by the SARA_p&g_ subscore [reprinted by permission from [36]]. Subgroups: CA_Mild_: SARA_g&p_ [0:2], CA_Mod_: SARA_g&p_= [3–4], CA_Sev_: SARA_g&p_ [5–6]. Shown are group differences for constrained lab-based walking and real-life walking). LatStepDev and the compound measure of spatial variability were sensitive in distinguishing these severity subgroups also during real-life walking

A first cross-sectional study on daily-life gait in degenerative cerebellar ataxia showed that it was feasible to measure in home environments and that stride-to-stride gait variability measures from inertial sensors — in spite of increased gait variability in real-life walking also in healthy participants [110] — demonstrate high sensitivity to small cross-sectional differences in disease severity, with higher effect sizes in daily-life walking compared to the SARA and clinical gait assessment [36]. Namely, *lateral step deviation* and a compound measure of spatial step variability (*SPcmp*) distinguished people with ataxia from healthy controls with a discrimination accuracy of 0.86. Both gait measures were highly correlated with clinical ataxia severity (SARA, effect size ρ=0.76) and patient-reported balance confidence (ABC-score, ρ=0.66). These measures detected group differences even when the difference was only 1 point in the clinical SARA_posture&gait_ subscore, with the highest effect sizes observed for real-life walking (effect size *d*=0.67, Fig. 7). The compound measure *SPcmp* — integrating variability in the anterior-posterior as well as in the medio-lateral dimension — hereby seems to benefit from capturing different compensation strategies employed in different disease stages.

A recent study examined ankle movements captured remotely by one IMU over a period of 1 week [73]. Individuals with ataxia revealed smaller, slower, and less powerful ankle submovements during natural behaviour at home. A composite measure based on ankle submovements strongly correlated with ataxia rating scale scores (Pearson’s *r* = 0.82–0.88), and self-reported function (*r* = 0.81) (PROM-Ataxia) [39], and had high test-retest reliability (ICC=0.95).

In addition to the analysis of straight walking episodes, analyzing turning movements in daily life distinguished not only ataxic, but also pre-ataxic, participants from healthy controls (effect sizes δ=0.68 and δ=0.53 respectively). Moreover, a measure of dynamic balance during turning detected a significant longitudinal change in a one-year follow-up assessment of people with degenerative cerebellar ataxia, with a large effect size (r_prb_=0.66) [38].

#### Challenges to Quantify Walking Behaviour in Daily Life

Despite these promising results, several challenges remain in recording gait in daily life. The total number of days or hours per day required to obtain reliable gait measures are uncertain. Studies in in other neurological diseases suggest that three days of monitoring may be sufficient to capture real-life gait performance; however, longer periods (e.g. 6 to 10 days) may be needed to fully capture day-to-day variability and establish strong correlations with patient-reported clinical measures [111].

In rea-life walking, gait measures are substantially influenced by contextual and environmental factors [112–114], both for healthy individuals and clinical populations (Parkinson’s disease, dementia, multiple sclerosis, cerebral palsy) [115–120]. Performance measures such as mean gait speed and especially gait variability measures are sensitive to gait bout length and other contextual factors [115, 118, 120] (e.g. stride length variability, stride duration variability). The analysis of shorter walking bouts for indoor walking — compared to longer walks outdoors — inherently delivers increased variability measures for both healthy controls and people with ataxia [110, 118]. Thus, it remains an open question whether the analysis should be restricted to a specific size of gait bouts, averaged over all gait bouts [115] or gait bouts matched according to macroscopic gait parameters [121]. Further work is required to determine the influence of data aggregation on real-world gait data in people with ataxia, particularly concerning the calculation of gait variability outcomes.

### Sensitivity to Longitudinal Change

**Fig. 8:**
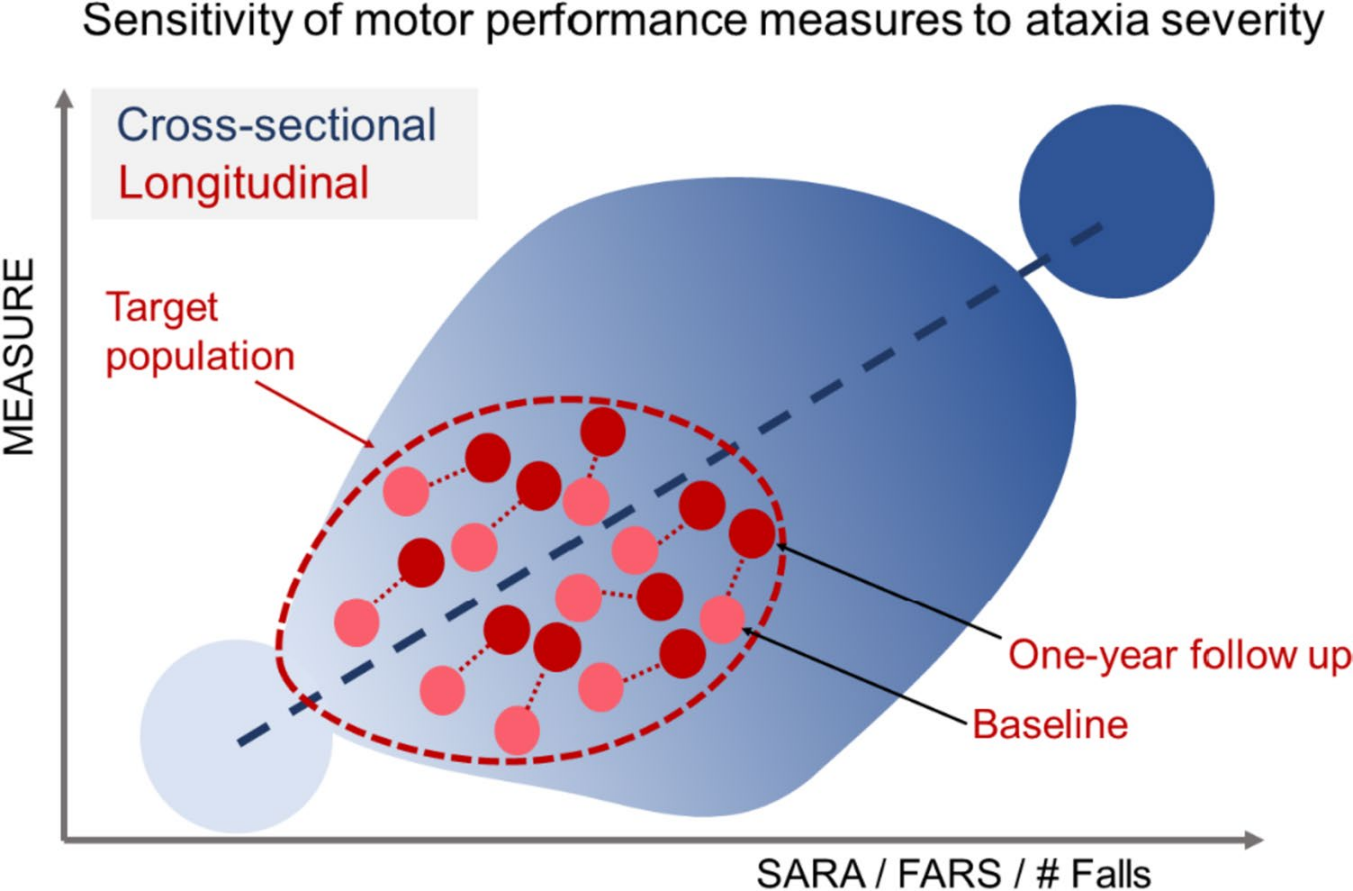
Illustration of different ways to show sensitivity to changes in ataxia severity. In most studies, **cross-sectional analysis** (blue) has been performed to show sensitivity to ataxia severity by correlations of balance and gait digital measures with clinical ataxia scores like the SARA, the FARS or the number of falls. These correlations with clinical ataxia scores are strongly influenced by the range of disease severity (range of observations [122]). **Longitudinal** (red): To serve as valid performance measure in ataxia intervention trials, these gait measures need to prove their sensitivity to individual longitudinal change over short time-spans (e.g. 1 year). In addition, the target population in clinical trials will most likely not encompass the full range of disease severity, but will be limited to, for example, mild-to-moderate disease

Gait variability and body sway measures have shown their sensitivity to ataxia severity predominantly via cross-sectional correlations with clinical ataxia scores [21–23, 29–31, 34, 36, 37, 50, 55–57]. However, these correlations with scores like SARA are strongly influenced by the range of disease severity (range of observations [122]) in the examined population. For cohorts that encompass a wide range of disease stages, many gait measures, including unspecific ones like gait speed, show significant correlation with disease severity, often predominantly driven by participants at the ends of the examined disease severity spectrum [122] (Fig. 8). Conversely, interventional trial requires the quantification of individual change in relatively short time-frames (e.g. over 1 year) in subjects with mostly mild-to-moderate disease (see 1-year follow-up in Fig. 8).

**Fig. 9:**
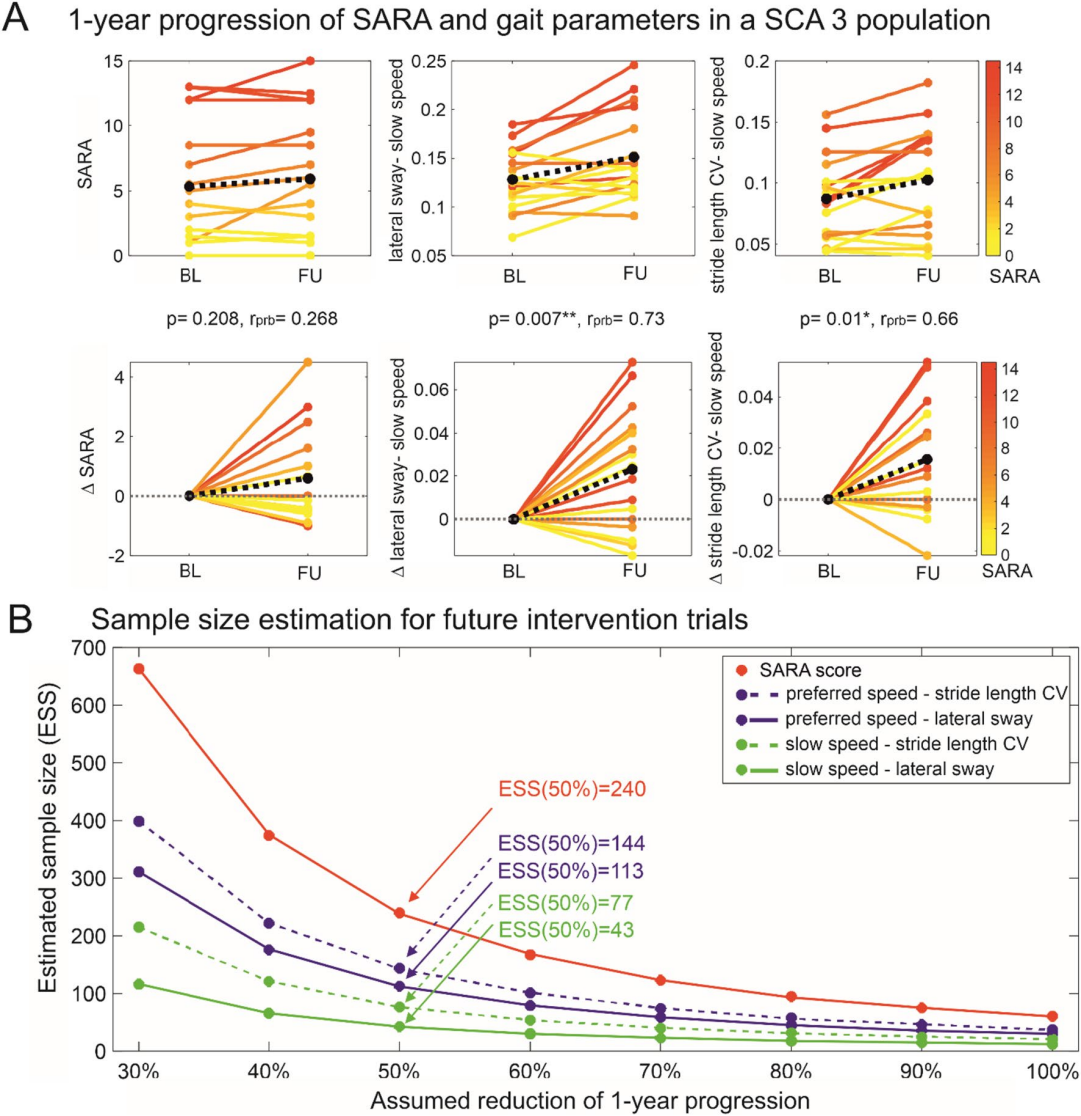
(A) Longitudinal analyses of 1-year follow-up assessments**:** Within-subject changes between baseline and 1-year follow-up for a SCA 3 group. Upper panel: Within-subject changes in the SARA score and the gait measures of lateral sway and Stride length CV in the slow walking condition from baseline (BL) at the 1-year follow-up (FU). Lower panel: Within-subject changes between baseline and 1-year follow-up represented as delta (∆). In all panels, SARA scores of individual participants with cerebellar ataxia are colour coded. Black dotted line = mean change across all participants. The stars indicate significant differences between timepoints (*≡ *p*<0.05, **≡ *p*<0.0083 Bonferroni-corrected, ***≡ *p*<0.001). Effect sizes r_prb_ were determined by matched-pairs rank biserial correlation. (B) Sample size estimations were performed for future intervention trials showing different levels of reduction in progression levels for the different outcome measures: SARA, lateral sway and stride length variability in the walking conditions with preferred and slow speed. The estimated number of participants per study arm is plotted over the assumed therapeutic effect for lowering the 1-year progression in SCA3 (reprinted from [25] with permission)

Few studies have examined the longitudinal course of gait impairment in ataxia, observing limited sensitivity to changes over time [61, 62, 123]. Changes in medial-lateral sway amplitude during gait using a triaxial accelerometer attached to the upper back were detected after 1.5 years in 25 people with spinocerebellar degeneration [65]. In a recent multicenter study based on 6 depth-imaging cameras (multi-Kinect system [35]), longitudinal analysis of 17 participants with SCA3 revealed significant change in gait measures between baseline and 1-year follow-up in slow gait with large effect sizes (stride length variability: r_prb_=0.66; lateral sway: r_prb_=0.73) (Fig. 9A) [25]. In this study, sample size estimation for lateral sway reveals a required cohort size of *n*=43 for detecting a 50% reduction of natural progression in this measure, compared to *n*=240 for the clinical ataxia score SARA (Fig. 9B).

A change over a 1-year follow-up was also identified in a mixed population of degenerative cerebellar disease for turning stability in daily life [38]. A measure that quantifies dynamic balance during turning — lateral velocity change (LVC) — detected of longitudinal change at 1-year follow-up (effect size: r_prb_=0.66). Larger multicenter longitudinal studies are needed to confirm and extend these findings and to specify gait, balance and turning metrics for more homogeneous ataxia populations [26, 121].

In FRDA, three studies have examined change due to natural disease progression over 12 months [62, 72, 124]. In the largest cohort of 52 participants, mean cadence (effect size SRM = −0.624) and velocity (SRM = −0.641) at fast speed were the most responsive spatiotemporal gait parameters and had larger effect sizes than the FARS, SARA [24] and mFARS [125]. Step width variability appears to be sensitive to FRDA disease progression, including children and those ambulating with and without an aid [62]. Knee extension range during stance also appears sensitive to disease progression in children [70], but it is yet to be seen if these changes continue in adulthood. In contrast to SCA3 [25], stride length variability at a self-selected speed seemed insensitive to disease progression in participants with FRDA [62, 70, 72].

## Recommended Consensus Protocols for the Assessment of Digital Gait and Balance Measures in Ataxia

In the following, we propose a digital gait and balance protocol for natural history studies and interventional trials in degenerative ataxias, on the basis of the (i) accumulated evidence on the sensitivity of gait and stance measures for quantifying ataxia (Tables 1 and 2) and (ii) established requirements for performance measures [126–131].

In developing such a protocol, it is important to consider that application is essentially confined to those able to stand and walk independently and that: (i) elementary gait and balance tasks may not be sensitive in early stages of ataxia; and, on the other hand, (ii) more complex gait and balance tasks may not be feasible for people with advanced ataxia. Therefore, we have divided the clinical gait and stance assessment protocol into a basic and a more complex part, with the latter which constituting tasks that are only suited for those participants in the earliest stages of the disease.

### Basic Protocol

Table 3 summarizes our recommendations for gait and balance assessments applicable to studies in individuals who are still able to stand and walk independently. Tasks with asterisks* indicate a minimum set. Participants should perform gait tasks (normal, comfortable pace and a self-determined ‘slow’ pace for 2 min over a 10-m pathway with 180° turns over a marker on the ground). Turns should be removed from the walking bouts for the gait analysis and analysed separately. Gait instructions and recommended details about the gait protocol can be found in the supplementary information.

**Table 3:**
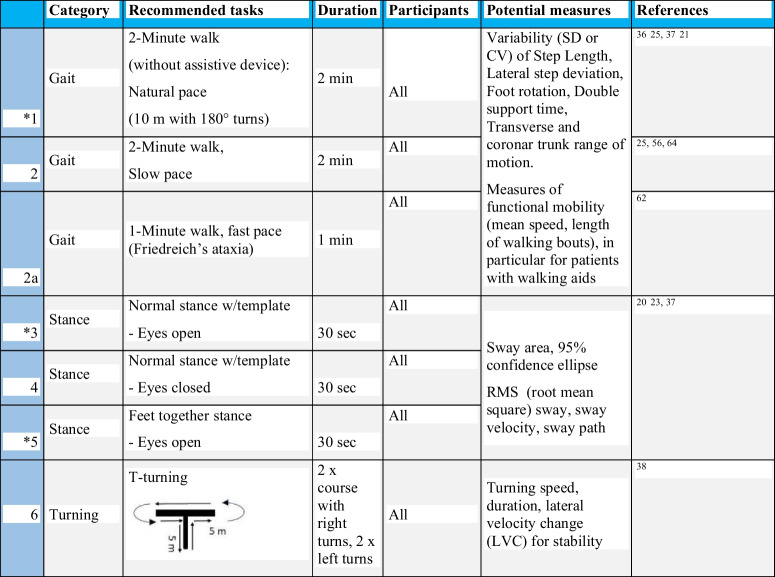
Proposed basic protocol for clinical assessment of gait and stance in ambulatory people with ataxia. Tasks with asterisks* indicate a minimum set. Natural stance assumes a fixed template for foot placement. Participants should not use any walking aid or assistive device if possible. Walking aids need to be documented. Gait tests with and without walking aids should not be mixed in analyses

Since gait variability is a sensitive and specific marker of ataxic gait, protocols should include at least 40 steps (20 strides) that have been shown to be required to ensure reliable measures of gait variability [132–134] and number of steps analysed should be reported along with results. As shorter distances intrinsically increase variability, the length of the walkway should be standardized in multicenter trials [16] [135] and is recommended to be 10 m.

We included slow-paced walking since slow walking has recently been shown to result in larger effect sizes of variability measures compared to natural paces walking for people with cerebellar ataxia [25, 56, 64]. These observations correspond with theoretical work on modelling human gait control, which postulates higher balance challenges with slow walking [136, 137].

However, 1 min of fast walking is recommended for sensory ataxias like Friedreich’s ataxia, as larger long-term effects have been observed compared to normal walking [62].

In general, recommendations of motor task and gait measures differ for individuals with FRDA compared with other hereditary (cerebellar) ataxias due to a number of factors. First, with the early onset of symptoms, on average 10–15 years of age [138], typical neural maturation, such as decreasing gait variability [139], changes in cognitive-gait interference [140] and sensory reweighting for postural strategies [141] impact gait and balance, concurrent with progression of ataxia. Second, time from symptom onset to wheel-chair dependency is only 10–15 years [142] with gait aid use as a common transition within this time. This provides a small window to include only independently-ambulant individuals in clinical studies, thus potentially necessitating the inclusion of participants who are dependent on walking aids (e.g. a cane or a 4-wheeled frame walker). Although the use of any assistive device or touching for stability makes it difficult to accurately interpret postural control in standing or gait variability in walking, the analysis of functional mobility (e.g. mean gait speed, mean length of walking bouts, see Table 4) in the course of disease may be useful for longitudinal studies.

**Table 4:** Proposed protocol for clinical assessment of gait and stance for pre-ataxic participants or those in the earliest stages of disease

	Category	Recommended tasks	Duration	Potential measures	References
7	Stance	Tandem stance - Eyes open	30 s	Sway area, RMS sway, sway velocity, sway path,	[20, 23, 37]
8	Stance	Feet together Stance - Eyes closed	30 s		
9	Gait	Tandem walk	2 x 8 m	Lateral trunk range, Stride Duration CV	[20, 23]

In addition to gait, standing balance should be assessed with 3 stance tasks (30 s each, eyes open and eyes closed with normal stance standardized with a template, as well as eyes open with feet together). Since people with ataxia tend to compensate for their large postural sway by widening their stance, protocols for measuring postural sway should be carefully controlled, settings standardised and stance width determined using a foot template [143]. The template we recommend is based on average, normal stance width in healthy young and elderly adults with 10 cm between the heels and 10° external rotation of the feet (15 cm wide at the toes) [144]. Since postural sway includes frequencies below 1 Hz, a minimum of 30 s of sway needs to be recorded [145].

In addition, we included a standardized turning task for the performance of 90° and 180° turns.

### Protocol for Early Disease Stages

The pre-ataxic phase of SCAs before the clinical manifestation of ataxia symptoms [8, 24] provides a promising window for early therapeutic intervention — both pharmaceutical and rehabilitative - before substantial, irreversible neurodegeneration has occurred [8, 146, 147]. Although some studies have identified gait and balance changes in pre-ataxic mutation carriers [22, 23], changes with larger effect sizes are found for more complex tasks such as tandem stance, tandem walking [20, 23, 51] and turning [38].

Thus, we have included more challenging gait and balance tasks for pre-ataxic or very mildly affected participants (Table 4). Participants are asked to perform a tandem walk and two additional stance tasks (eyes closed with feet together and tandem stance with heel touching opposite toe with eyes open).

In addition to the clinical assessments of prescribed gait and balance tasks we also propose continuous monitoring of gait and turning during daily life for 7 days (minimum 5 hours per day) for both pre-ataxic and manifest ataxia (Table 5).

**Table 5:** Monitoring of walking behaviour during daily life

	Category	Recommended tasks	Duration	Participants	Potential measures	References
10	Daily-Life monitoring	Daily life monitoring of walking and turning including indoor and outdoor motion	7 days	All ambulatory	Average gait speed, length of walking bouts, Lateral step deviation, Stride length CV in gait, LVC in turning	[36, 38]

### Test-Retest Reliability and Minimal Detectable Change

Studies of gait and balance ataxia should repeat tests at baseline. Useful gait and balance outcomes need to demonstrate stability of measures over time when no change is expected, such as in re-test within a short timeframe. Test-retest reliability can then be calculated using intraclass correlation coefficients (ICCs) [148] and Bland-Altmann Plot with limits of agreement [149, 150].

One way to investigate the reliability (in terms of technical repeatability) of the gait measures is to divide a 2 minute-walk test into two, 1-minute segments, and calculate the split-half reliability of gait measures ICC (2, 1) [148]. As ataxia can show considerable day-to-day fluctuations [151], a more rigorous way to calculate test-retest reliability is to have the participants repeat the test twice, after a period of rest or on another day.

In a study examining gait measures from the 2-minute walk test, the within session test–retest reliability of the most sensitive/specific gait variability measures discriminating participants with SCA from healthy controls was good-to-excellent (0.83–0.9) [21]. Moreover, high test-retest reliability was reached for ankle movement measures in real life by comparing data recorded over days 1–3 and days 4–6 [73].

Based on the test-retest reliability measured by the ICC, the minimal detectable change (MDC) can be determined. MDC indicates the minimum change that falls outside the measurement error and can be statistically detected with some degree of confidence (e.g. 95 or 90%) from a test-retest reliability design [152–154].

$$MDC=z-\textrm{scor}{e}_{\textrm{level}\ \textrm{of}\ \textrm{confidence}}\times S{D}_{\textrm{baseline}}\times \left(\sqrt{2\left[1- ICC\right]}\right).$$

This value is a fundamental technical metric that indicates the “noise” above which a change can be considered beyond technical error and potentially daily fluctuations. The ability of a measure to detect change over time in this sense has been referred to as internal responsiveness of an outcome [155].

## Recommended Recording Technology

While sensitive digital gait measures have been identified using a variety of recording technologies (Reviews in [16, 18, 28, 109]), not all are equally suitable for multicentre clinical trials that need to include centers without a dedicated motion laboratory or specialised technical staff (see Table 6 for comparison of recording technologies in clinical trials). While laboratory-based, optical motion analysis systems remain the gold standard for gait analysis, they are expensive, resource intensive, and largely immobile, which limits their accessibility in clinical settings [135, 156]. Other cost-efficient camera-based systems (e.g. Kinect) often have limitations in the recording space, which reduce the length (≤5 m) of the captured walkway important for gait variability.

**Table 6:** A summary of the advantages and disadvantages/challenges of recording technology used to quantify gait and postural control. Adapted and extended from [109]

Device	Advantages	Disadvantages/challenges
3D optical motion capture (e.g. Vicon)	- Considered as gold standard - Highly precise and accurate - Potential to measure a large variety of outcomes (e.g. including joint angle trajectories) - High-resolution data	- High cost - Requires experienced technical expertise - Requires a large purpose-built dedicated space usually limited to laboratory/research environments - Participant preparation can be time-consuming
Force plates	- Considered gold standard for measuring ground reaction forces and centers of pressure - Minimal space required - Minimal participant preparation time - High-resolution data	- High cost - Requires experienced technical expertise - Requires a purpose-built dedicated space - Can capture only single steps
Instrumented mats (e,g. GAITRite)	- Minimal processing time - Minimal participant preparation time - Portable	- Calculable features are limited by mat dimensions - Requires a large space to accommodate the mat dimensions - Limited to temporal spatial and foot pressure gait outcomes of the lower extremities
Depth-camera-based (e.g. Kinect)	- Low-cost - Whole body kinematics - Minimal participant preparation time - Portable	- Often limitations of captured walkway (≤5 meters) - Challenges in identifying exact step events
Inertial measurements units (IMUs)	- Capable of capturing continuous movements in laboratory and community environments without space limitations - Minimal preparation time - Certain systems provide automated reports - Cheaper than the gold standard - Portable, easy to use in commercial systems	- Requires dedicated algorithms and expertise to calculate key features - Features are often indirect measures requiring additional participant measurements - some restrictions on available measures (e.g. joint angles) - Measures depend on sensor configuration - Free-living measurements may be limited by recording time or data storage

Given the described influences of walkway length on step variability measures, it is important to consider the influence of technical restrictions of the equipment on study design, gait protocol and parameter definitions [16, 109].

Wearable IMU sensor technology for quantifying gait and balance has recently become feasible for large, multicenter clinical trials without sophisticated gait laboratories or expert researchers.

These body-worn sensors enable the recording of longer gait distances, are portable and instantly provide gait metrics without post-trial human analysis, making IMUs are easy to use in clinical settings. Moreover, they wearable IMUs allow movement monitoring in daily life [36, 157, 158] (Reviews in [109, 159]). Therefore, wearable sensor technology has been identified as the most appropriate technology at this time to conduct such multicenter studies of digital gait and balance measures in ataxia [127, 160].

However, IMUs require specific algorithms [161–163] and technical verification of the accuracy of the hardware, raw signals and software (with the algorithms producing the gait and balance metrics) [161163]. Sensors employed for clinical trials must be validated by comparison with laboratory gold-standards [160]. For example, a study of SCA14 showed good-to-excellent between-methods consistency with APDM’s Opal sensors (using Mobility Lab) and measures of mean gait metrics derived from GAITRite, with ICCs >0.9, except for stride length (ICC=0.84) [33].

The optimal number of sensors in the trade-off between gait data quality and participants burden currently appears to be three: one sensor at the lower lumbar spine, and one sensor on the top of each foot. This configuration has substantial advantages for the quantification of ataxic foot placement characteristics, compared to only one sensor on the lumbar spine, as is widely used for activity-monitoring [117, 164, 165]. First, several measures showing the best sensitivity to ataxia require sensors on the feet (e.g. lateral step deviation and pitch angle at heel strike) [21, 36]. Second, in order to accurately measure step variability (e.g. variability in stride length and stride duration), accurate determination of step events (initial and final foot contacts) is crucial. At least current recording techniques and algorithms using only one IMU or mobile phone at the pelvis show limited accuracy and reliability of gait variability measurements, which is probably low due to inaccurate identification of heel strike and toe-off events [57, 166, 167] (see review in [135]). In addition to the IMUs on the feet, the lumbar sensor provides measures of trunk instability (range of motion and jerkiness of the trunk), as well as measures of turning (turn velocity, duration, etc.). Postural sway in standing can also be measured with an IMU on the lumbar spine, but recent studies suggest that even more sensitive measures of ataxic sway can be obtained from an IMU on the sternum [85, 168].

Future promising developments in recording technology could include the combination of IMUs with pressure sensitive insoles [169] as well as video-based technologies using machine learning methods to generate specific movement features (see for review [170]).

## Recommended Inclusion/Exclusion Criteria

The following inclusion criteria for establishing digital balance and gait metrics are recommended as follows: (1) genetically confirmed hereditary cerebellar ataxia; (2) degenerative cerebellar ataxia in the absence of any unrelated signs of other CNS disease; (3) age between 18 and 80 years for SCAs. For Friedreich’s ataxia, younger participants are required due to the earlier onset: age between 8 and 65 years (see [62]); (4) able to walk for 2 min and stand for 30 s with eyes open, without walking aids. The exclusion criteria should include the following: (1) severe visual or hearing disturbances, (2) cognitive impairment limiting ability to follow protocol instructions, (3) orthopaedic or unrelated neurological constraints affecting standing and walking, and (4) drug or alcohol history which related to ataxia.

If participants who require walking aids for the 2-minute walk are recruited, they must be analysed separately, and the use of an assistive device needs to be documented. Natural history studies should also include age-and sex-matched healthy control subjects without known neurological or musculoskeletal impairments that affect balance or walking.

## Recommended Clinical Assessments and Patient-Reported Outcomes

Every natural history study of ataxic gait and balance should include clinical measures of ataxia symptoms, such as the SARA [24] for SCA, mFARs [125] for Friedreich’s Ataxia; the INAS for other non-ataxia symptoms, and patient-reported outcomes including the new PROM-Ataxia [39], EQ-5 [45], and the Activities-specific Balance Confidence (ABC) Scale [107] (See Table 7). The most sensitive measures of gait and balance should then be correlated with clinical assessment of severity of ataxia [24] for Concurrent Validity (see Figs. 1, 2, 4) as well as with patient-reported scales for meaningfulness to people with ataxia [39, 107] (see also the AGI Consensus Recommendations for Clinical Outcome Assessments [171]).

**Table 7:** Overview of the most important clinical ataxia scales, questionnaires of quality of life and patient reported-outcomes measures which should correlated with gait and balance performance measures in order to show concurrent validity and meaningfulness of gait and balance measures to the daily lives of people with ataxia

Outcome	Measure	Description
Ataxia symptoms	SARA [24] or mFARS [125]	The SARA is a clinical assessment of ataxia, measuring upper limb, lower limb, gait, balance and speech. Eight items; score range 0–40, with a higher score indicating more severe ataxia [24]. The mFARS (modified Friedreich’s ataxia rating scale) is a clinical assessment of Friedreich’s Ataxia ranging from 0 (no ataxia) -93 (severe ataxia). 18 items grouped into 4 sub components: bulbar, upper limb co-ordination, lower limb co-ordination, upright stability [125].
Non-ataxia symptoms	INAS [180]	INAS (Inventory of Non-Ataxia Signs) provides structured information on non-ataxia signs in people with ataxia. Consists of 30 items, related to one of 16 non-ataxia signs (e.g. areflexia, hyperreflexia, spasticity, paresis, amyotrophy, fasciculations, myoclonus, rigidity, chorea, dystonia, resting tremor, sensory symptoms, brainstem oculomotor signs, urinary dysfunction, cognitive impairment).
Balance confidence	ABC [107]	Activities-specific balance confidence (ABC) scale is a structured questionnaire that measures an individual’s confidence during ambulatory activities. It consists of 16 questions gauging the individual's confidence while doing activities.
Activities of daily living (ADL)	FARS-ADL [181]	The FARS ADL assesses an individual's ability to perform various activities of daily living, such as dressing, grooming, bathing, toileting, feeding, and mobility. The scale ranges from 1 to 7, with higher scores indicating greater independence in performing daily activities. A score of 1 indicates complete independence, while a score of 7 indicates complete dependence.
Patient-reported outcome measures (PROM)	PRoM-Ataxia [39]	The PROM-Ataxia is a 70-item questionnaire emerging from lived experience. It includes questions about gait, lower and upper extremity control, manual dexterity, visual/ocular motor control; patient experience of dysphagia, bowel and bladder function, sleep, fatigue, vertigo, neuropathy, ability to manage household chores and employment responsibilities, driving, libido and self-care; around 20 items include aspects of gait and balance.
Patient global impression (PGI) of severity and change	PGI-S, PGI-C [177, 178]	Patient global impression of severity (PGI-S): Rate the severity of your disease right now: 1: not present, 2: very mild, 3: Mild, 4: Moderate, 5: Moderately severe, Severe, 7: extremely severe Patient global impression of change (PGI-C): Since the last visit, my overall status/functional mobility/gait & balance has: 1: very much improved, 2 much improved, minimally improved, 4: No change 5: minimally worse, 6: much worse, 7: very much worse

Avoiding falls is also meaningful to people with ataxia [31, 172] so longitudinal studies should include prospective monitoring of falls over time using recommended guidelines [173, 174]. Falls can be defined as a “sudden, unintended contact with the ground” and should be queried 1–2 times per month via email or texts for accurate recall, with phone follow-ups if a fall occurred or if the queries are not answered. Gait and balance measures that predict falls or separate fallers from non-fallers can help determine which measures are meaningful to people with ataxia.

To quantify progression and treatment responses, gait and balance measures should capture longitudinal changes that correspond to minimal clinically important differences (MCID) [175] and functional changes in patient-centred outcome measures [1, 160, 176]. MCID for how much a balance or gait measure needs to change for a person with ataxia to perceive a small difference can be captured by asking them to rate their change in balance or gait (after longitudinal progression or treatment) on a 7-point Likert scale [177, 178] (Patient global impression of change (PGI-C), see Table 7) [179]. The Likert scale has 3 points indicating worse balance/gait and 3 points indicating better balance/gait with 0 indicating no change. A MCID would correspond to a patient reporting a +1 or −1 on the Likert Scale [177]. Of course, difficulties of recollection of one’s own balance and gait impairments in the past 6–12 months is a limitation of this approach. By concept, the choice of MCID anchor may differ between contexts of use [179].

## Regulatory Considerations for Digital Gait and Balance Outcomes for Clinical Trials

The regulatory pathway for including endpoints derived from body-worn sensors varies across regulatory agencies.

The FDA has a program for Clinical Outcome Assessments (COAs), that are the most suitable for gait and balance quantification for clinical trials and that are not specific to a single, individual drug development program [131, 179]. COAs are defined as measures that reflect how a person feels, functions, or survives. The FDA has specified five different types of COAs. Measures from body-worn sensors during prescribed tasks, such as walking and standing, would be considered as *performance outcomes* (PerfO). Many criteria are considered as part of the qualification process including those described here, such as reliability, validity, and meaningfulness [160] .

**Fig. 10:**
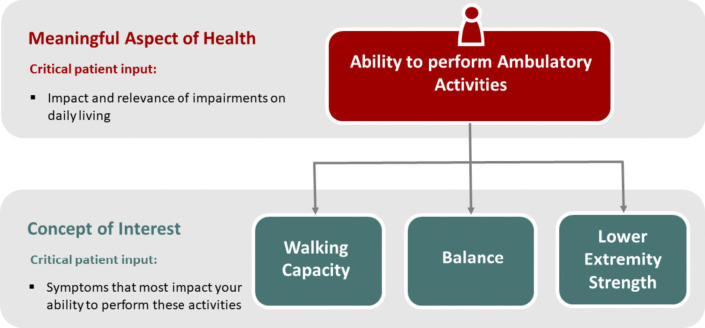
Examples of how a variety of concepts of interest cascade from a single meaningful aspect of health across select conditions and clinical populations (adapted from [182])

In particular, trial endpoints must first be based on a meaningful aspect of health, such as ability to perform ambulatory activities, from which various concepts of interests, such as walking and balance cascade. Figure 10 illustrates examples of these concepts [182].

As described above, it is well known that impairments in gait and balance due to ataxia are some of the most important symptoms, as they have a direct impact upon the quality of life of people with ataxia [40–42] and their ability to perform normal daily activities independently [12, 13]. While there are now patient-oriented measures that address gait and balance impairments in daily life (PROM-Ataxia, ABC-score [39, 107]), there is yet no study showing their longitudinal sensitivity. When such studies have been completed, changes in gait and balance measures should be interpreted against changes in patient-reported outcomes. Moreover, gait and balance measures, and especially how they change over time, need to be linked to meaningfulness in different stages of the disease.

One possible approach for such a link is to establish Meaningful Score Regions (MSR) or Meaningful Score Changes (MSC) for gait and stance metrics by relating them to different levels of gait-related Patient Global Impression of Severity or Patient Global Impression of Change (see Table 7) [179]. However, it is not yet exactly clear, which specific aspects of functional mobility should be rated relative to specific gait and balance metrics: e.g.: mobility, walking quality, postural stability, unsteadiness, fear of falling, gait variability.

Another issue that has not yet been fully resolved arises from the problem that while a discrete increase in gait variability does not noticeably affect a person with ataxia in the early stages of the disease, a gradual increase in gait variability leads to a much higher risk of falls later in the course of the disease [31, 183]. Thus, a therapy-induced reduction in gait variability in the earlier stages of the disease may have a significant impact on later disease progression and thereby prolonging independent ambulation.

Therefore, evidence is needed on what changes in gait and balance performance measures are meaningful for people with ataxia not only in their current condition, but also as the disease progresses over time.

## Summary

The tasks recommended in this consensus protocol are strongly associated with typical daily activities that people perform regularly. Standing, walking and turning are performed throughout the day and are critical functional tasks in daily life. These are also activities that are impaired early in ataxia and are frequently identified as the most disabling and thereby meaningful and relevant to daily functioning.

Digital gait and standing balance measures represent promising endpoints for upcoming interventional trials. Our consensus recommendations to allow multisite pooling of natural history data on gait and balance impairments for ataxia include:

a) *Protocols:* Include a minimum of a 2-minute walk (use a walkway with the standardized length of 10 meters) and a 30-second standing task with additional conditions or greater challenge for pre-ataxic ataxia;

b) *Recording Technology*: 3 body-worn inertial (IMU) sensors (one at the pelvis or sternum and two on the feet);

c) Sensitivity/Specificity: Calculate AUC of ROC curves to identify gait and balance measures that best separate individuals with ataxia from age-matched controls;

d) *Test-retest reliability:* Show test-retest reliability and calculate a *Minimal Detectable Change* (MDC);

e) *Meaningfulness:* Calculate *Minimal Clinically Important Change* MCID for sensitive digital measures by including a patient-reported scale of perceived change;

f) *Concurrent Validity:* Include standard neurological scales of severity (e.g. SARA or mFARS);

g) *Longitudinal assessment of natural course:* Test participants every 6 months for 2 years. Demonstrate longitudinal changes over a reasonable study period (e.g. within 1–2 years) to enable sample size estimation for future clinical trials.

h) *Daily life:* monitoring of walking behavior over 7 days of daily life (for a minimum of 5 h daily), with the same IMU system.

Based on our current knowledge, the same gait and balance digital outcomes can be used for a range of cerebellar ataxias, with the most evidence currently available on the most common SCA including 1, 2, 3 and 6 (see Fig. 1B and [21]). In Friedreich’s ataxia, the gait measures with the greatest sensitivity to longitudinal changes may vary somewhat due to the predominance of sensory ataxia, the younger age range during adolescence, and the more rapid dependence on walking aids.

In general, the most sensitive tasks and measures will likely depend primarily on the disease stage of the targeted trial population. More complex movement tasks, such as tandem walking, tandem stance or eyes-closed stance are proposed for pre-ataxic and early ataxic participants. Gait variability over a natural-pace 2-minute walk and postural sway area during a feet-together, eyes-open stance for 30 s are currently the most promising outcomes for prescribed tasks in the clinic/laboratory. Although active monitoring of prescribed gait and standing tasks currently provide the most reliable data, daily life monitoring holds great promise for providing even more meaningful measures of actual functional mobility. The goal is to identify the most sensitive gait and balance measures, or a composite measure, that has a larger effect size than current clinical scales to detect disease progression so that clinical trials for these rare diseases could be conducted with smaller cohorts.

## Recommendations for Further Studies

Existing studies have provided significant evidence that digital gait and balance measures can be sensitive performance markers for ataxia with excellent reliability and validity characteristics. In addition, ataxia-related gait changes are related to meaningful aspects of health, e.g. a high risk of falls is associated with increased gait variability and increased postural sway. However, we need to establish the meaningful score difference for digital gait and balance outcomes by relating them to patient global impression of severity (PGI-S) or change (PGI-C) [178, 179]*.* Furthermore, future studies should compare active versus passive monitoring of gait as useful outcomes for ataxia, as well as explore whether reliable measures of gait can be obtained from individuals using walking aids.

There is a consensus that a large, international effort to collect digital balance and gait measures longitudinally is necessary (such as performed in other neurodegenerative diseases like Parkinson’s disease [184]). This data, and in general as much data as possible collected through the proposed consensus protocol, should be openly available to support the further development of digital gait and balance outcomes for ataxia clinical trials.

### Supplementary information

ESM 1
