# Supplementary material for: Quantitative Gait and Balance Outcomes for Ataxia Trials: Consensus Recommendations by the Ataxia Global Initiative Working Group on Digital-Motor Biomarkers
Source: Cerebellum. 2023 Nov 13;23(4):1566–92. doi: 10.1007/s12311-023-01625-2 (PMC11269489; doi:10.1007/s12311-023-01625-2)
Supplement: Supplementary file 1 — ESM 1 [file 12311_2023_1625_MOESM1_ESM.pdf]

Supplement: Instructions to gait & balance tasks

- Both gait and balance should be evaluated with the participants wearing comfortable, athletic shoes but not shoes with high heels, boots, sandals, slippers, etc.
  - Gait and stance tests with shoes instead of bare feet are recommended for (i) safety reasons and (ii) higher relevance for the daily lives of people with ataxia.
- The space for the standing and walking task should be well lit and without distractions, such as others walking nearby.
- Minimal width of the walkway should be 2 meters
- The wall should not be within reach of the participant during the gait test
- Participants should not use any walking aid if possible, walking aids have to be documented. Gait tests with and without walking aids should not be mixed in analyses
- If necessary for safety, a study assessor should follow the participant
- It is highly recommended to video record the gait tests – video from frontal plane, use a tripod

Tables S1, S2 and S3 describe the detailed instructions for motor tasks and the daily life monitoring.

Table S1 Basic protocol of recommended tasks and task instructions in balance and gait.

	Category	Recommended Tasks	Duration	Instructions
*1	Gait	2-Minute Walk :<br>Natural pace<br>(10 m with180° turns)	2 min	Participants are instructed to walk at preferred speed (i.e., a pace they deemed as comfortable or normal), as long as possible up to 2 minutes. When they reach the line at the end of the 10 meters walkway, they should try not to stop and simply turn around and walk back.
2	Gait	2-Minute Walk<br>Slow pace	2 min	Participants are instructed to walk at a slower pace (instruction: walk with slower gait speed than normal), as long as possible up to 2 minutes.
*3	Stance	Normal Stance w/template<br>- Eyes open	30 sec	The participant should stand on a firm surface, with their feet apart, eyes open, and their hands on their side. Participants should look straight ahead and remain still without talking or moving. To normalize their stance, place a foot template (see text) on the ground and adjust the stance.
4	Stance	Normal Stance w/template<br>- Eyes closed	30 sec	The participant should stand on a firm surface, with their feet apart, eyes closed, and their hands on their side. Participants should look straight ahead and remain still without talking or moving. To normalize their stance, place a foot template on the ground and adjust the stance.

*5	Stance	Feet together Stance<br>- Eyes open	30 sec	The participant should stand on a firm surface, without shoes, with feet together in parallel (big toes and heels touching each other), eyes open, and their hands on their side.
6	Turning	T-Turning<br>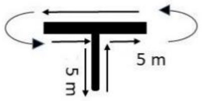	2 x right turns,<br>2 x left turns	Participants are instructed to walk along a path at a T-junction of corridor to perform 90° and 180° turns as illustrated, supervised by a study assessor. This task should be conducted twice in each direction (1-2 min).

Table S2 Protocol of additional recommended tasks and task instructions in pre-ataxic and early phases of cerebellar ataxia.

	**Category**	**Recommended Tasks**	**Duration**	**Instructions**
7	Stance	Tandem Stance<br>- Eyes open	30 sec	Participants are instructed to stand quietly with your feet in tandem (no space between heel and toe) and your hands at your side, facing straight ahead
8	Stance	Feet together Stance<br>- Eyes closed	30 sec	The participant should stand on a firm surface, without shoes, with their feet together in parallel (big toes touching each other), their eyes closed, and their hands on their side.
9	Gait	Tandem Walk	2 x 8 m	Participants are instructed to walk by placing one foot in front of the other (no space between heel and toe)

Table S3 Instructions for the daily life monitoring of walking and turning

	**Category**	**Duration**	**Instruction**
10	Daily life monitoring of walking and turning	7 days	Participants should be instructed to wear the foot sensors for the entire day for at least 5 days during their normal daily activities. The recordings should include bouts of indoor and outdoor walking. Participants should maintain an activity diary.<br><br>If mobility activity level is of interest (i.e.; number and length of gait bouts, number of turns, etc.), a weekend should be included. Participants should indicate in the activity diary any walks that include an assistive device so they can be analyzed separately. Since daily life walking bouts are generally much shorter (ie; 2-10 strides) than prescribed tests in the clinic, comparison of gait characteristics during daily life versus in the clinic should include similar stride lengths[115, 121]. Inertial sensors should be located both on the feet or ankles to monitor gait variability, as well as on the trunk (Lumbar area to measure trunk stability and turning quality.
